# Reactive oxygen species inhibit catalytic activity of peptidylarginine deiminase

**DOI:** 10.1080/14756366.2017.1368505

**Published:** 2017-09-21

**Authors:** Dres Damgaard, Mads Emil Bjørn, Peter Østrup Jensen, Claus Henrik Nielsen

**Affiliations:** a Institute for Inflammation Research, Center for Rheumatology and Spine Diseases, Copenhagen University Hospital, Rigshospitalet, Copenhagen, Denmark;; b Section for Periodontology, Microbiology and Community Dentistry, Department of Odontology, Faculty of Health and Medical Sciences, University of Copenhagen, Copenhagen, Denmark;; c Department of Haematology, Roskilde Hospital, Roskilde, Denmark;; d Department of Clinical Microbiology, Copenhagen University Hospital, Rigshospitalet, Copenhagen, Denmark

**Keywords:** Peptidylarginine deiminase, citrullination, enzymatic activity, reactive oxygen species, NADPH oxidase, hydrogen peroxide, arthritis

## Abstract

Protein citrullination catalysed by peptidylarginine deiminase (PAD) may play an important pathogenic role in several chronic inflammatory diseases and malignancies. PAD2, PAD4, and citrullinated proteins are found in the synovium of rheumatoid arthritis patients. PAD activity is dependent on calcium and reducing conditions. However, reactive oxygen species (ROS) have been shown to induce citrullination of histones in granulocytes. Here we examine the ability of H_2_O_2_ and leukocyte-derived ROS to regulate PAD activity using citrullination of fibrinogen as read-out. H_2_O_2_ at concentrations above 40 µM inhibited the catalytic activity of PAD2 and PAD4 in a dose-dependent manner. PMA-stimulated leukocytes citrullinated fibrinogen and this citrullination was markedly enhanced when ROS formation was inhibited by the NADPH oxidase inhibitor diphenyleneiodonium (DPI). In contrast, PAD released from stimulated leukocytes was unaffected by exogenously added H_2_O_2_ at concentrations up to 1000 µM. The role of ROS in regulating PAD activity may play an important part in preventing hypercitrullination of proteins.

## Introduction

Citrullination, i.e. conversion of peptidyl-arginine into peptidyl-citrulline, is a post-translational protein modification catalysed by peptidylarginine deiminase (PAD) in a calcium-dependent manner^1^. Five isoforms of PAD exists^2^, of which PAD2 and/or PAD4 are thought to play important pathophysiological roles in chronic inflammatory diseases such as RA^3^, multiple sclerosis and type 1 diabetes^4–6^, as well as in certain forms of cancer^7^.

In addition to calcium, the catalytic activity of PAD requires reducing conditions^8^. These are usually obtained *in vitro* using the non-physiological reducing agent dithiothreitol (DTT). We demonstrated recently that reduced glutathione (GSH), at low millimolar range corresponding to those found intracellularly^9^^,^^10^, can substitute for DTT as PAD-activating reducing agent^11^. Moreover, we found that PAD released from phorbol 12-myristate 13-acetate (PMA)-stimulated leukocytes was capable of citrullinating fibrinogen in the vicinity, with granulocytes being the predominant source of active PAD^11^. However, PAD contained in cell supernatants was inactive, which lead us to hypothesize that it was rapidly oxidized.

Activated neutrophils produce superoxide radical (O^•^_2_^−^) catalysed by the nicotinamide adenine dinucleotide phosphate (NADPH) oxidase 2 (NOX2)^12^^,^^13^. Consequently, other reactive oxygen species (ROS) are formed, including hydrogen peroxide (H_2_O_2_), hydroxyl radicals, and hypochlorous acid^14^^,^^15^. ROS can be released from neutrophils in response to strong stimuli such as PMA, or the physiological secretagogues, including immune complexes, C5a, N-formylated peptides, leukotrienes, platelet-activating factor, and arachidonic acid^16–22^. Intracellular ROS may promote intracellular histone H3 citrullination^23^^,^^24^ and NETosis^23^^,^^25^, which has led to the suggestion that ROS may activate PAD4 downstream^26^. However, such studies have generally not concerned extracellular proteins, which account for a large part of citrullinated proteins in the synovium of RA patients^27^^,^^28^.

Here, we examine the direct effect of H_2_O_2_ on the activity of recombinant human PAD2 and PAD4, and on the extracellular citrullination mediated by PAD released from PMA-stimulated leukocytes. Moreover, the role of NOX2 in regulation of extracellular PAD activity is examined.

## Experimental procedures

### Cells

Blood samples were drawn into 9 ml lithium-heparin tubes (BD Bioscience, Brøndby, Denmark) from healthy donors attending the Blood Bank at Copenhagen University Hospital, Rigshospitalet, Denmark. The National Ethical Committee of Denmark waived the need for ethical approval for this study due to the anonymity of the donors included. Pooled serum from blood group AB-positive donors, henceforward referred to as “AB serum”, was purchased from Sigma-Aldrich (St Louis, MO). Leukocytes were isolated after lysis of erythrocytes by incubation with ammonium chloride buffer (In Vitro As, Fredensborg, Denmark) for 7 min in the dark. Upon lysis, cell were centrifuged at 300 *g*, at room temperature for 5 min and washed twice in RPMI 1640, 25 mM Hepes containing 0.42 mM calcium nitrate, l-Glutamine and Gentamicin (In Vitro As, Fredensborg, Denmark).

### Reagents

Recombinant human PAD2 (rhPAD2) and rhPAD4 were produced and purified as described previously^29^. GSH, diphenyleneiodonium (DPI), PMA, fetal calf serum (FCS), and H_2_O_2_ were purchased from Sigma-Aldrich. Dihydrorhodamine-123 (DHR-123) and near-infra-red (NIR) were purchased from Molecular Probes (Life Technologies Europe BV, Naerum, Denmark). Monoclonal mouse anti-citrullinated fibrinogen antibody (clone 20B2) was purchased from ModiQuest (Oss, Netherlands). Allophycocyanin (APC)-conjugated anti-CD15 antibody was purchased from BD Bioscience (Albertslund, Denmark).

### Assay for PAD activity

As described^11^, Maxisorp plates (Nunc, Roskilde, Denmark) were coated overnight at 4 °C with 1 µg/ml fibrinogen (Calbiochem, Darmstadt, Germany). Wells were washed thrice and blocked in Tris-buffered saline (TBS) buffer containing 0.05% Tween-20, pH 7.4. Hereafter, the wells were incubated for 3 h at room temperature (RT) with PAD-containing samples with or without GSH, H_2_O_2_, and EDTA, as stated in figure legends. Purified leukocytes were applied in RPMI 1640 containing 5% AB serum and, when relevant, 15 nM PMA, 25 µM DPI, which inhibits NOX2 and other flavoenzymes by interacting with the flavin part^30^, or H_2_O_2_ at various concentrations. After three washes in washing buffer (PBS, 0.05% Tween-20, pH 7.4), mouse anti-citrullinated fibrinogen antibody (0.5 µg/ml) and hereafter horse radish peroxidase-conjugated polyclonal rabbit-anti mouse immunoglobulin antibodies (P0260, Dako, Glostrup, Denmark) were added and developed as described^11^. Optical density (OD) was measured at 490–650 nm using the SPECTROstar nano Microplate Reader (BMG Labtech, Ortenberg, Germany). Data were processed using MARS software (BMG Labtech).

### Measurement of PAD2 concentration

PAD2 was measured using an in-house luminex based sandwich immunoassay using two specific anti-PAD2 antibodies previously used in an ELISA^31^. The lower limit of detection of PAD2 was 100 pg/ml using rhPAD2 as calibrator. Cell supernatants were diluted 1:1 with PBS containing 0.05% Tween-20, pH 7.4.

### Flow cytometric assessment of H_2_O_2_ formation by granulocytes

Isolated blood leukocytes from 9 ml blood were resuspended in 9 ml RPMI containing 10% AB serum. DHR-123 was added to all the tubes to a final concentration of 1.5 µM. Half of the samples received 25 µM DPI. Each group of samples were incubated with H_2_O_2_, at various concentrations, or were stimulated with 30 nM PMA or left unstimulated for 60 min at 37 °C, 5% CO_2_. After wash in RPMI, the cells were stained incubated for 40 min at RT with APC-conjugated anti-CD15 antibody for identification of live neutrophils, and LIVE/DEAD^®^ Fixable Near-IR (NIR) Dead Cell Stain (Molecular Probes, Eugene, OR) in order to discriminate between live and dead cells. Cells were subsequently acquired on a FACS-CANTO-II Flow-cytometer (BD Bioscience) and analysed using Flow-Jo software (version 10, Ashland, OR). The amount of H_2_O_2_ in the granulocytes was expressed as the median fluorescence intensity within the FITC-channel (FL-1) of live (NIR-negative) granulocytes, defined on the basis of morphological forward light scatter (FSC)/side light scatter (SSC) gate and positivity for CD15-expression.

### Statistics

Wilcoxon matched-pairs signed rank test was applied to compare PAD activity or PAD2 release in the presence and absence of DPI. To this end, the GraphPad Prism 7.02 software (GraphPad, San Diego, CA) was employed. Two-tailed *p* values < .05 were considered significant.

## Results

### Reactive oxygen species inhibit PAD activity

**Figure 1.**
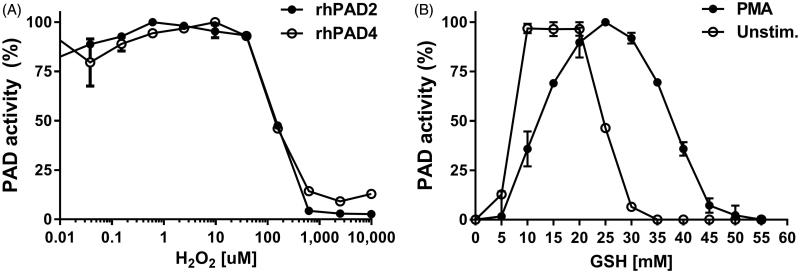
Effect of ROS on PAD activity. (A) Microtiter wells were coated with human fibrinogen and incubated for 3 h at room temperature with rhPAD2 (300 ng/ml) or rhPAD4 (3000 ng/ml) in 100 mM Tris-HCl including 15 mM reduced glutathione (GSH) and 5 mM CaCl_2_. (B) Supernatants from unstimulated leukocytes or leukocytes stimulated with PMA for 30 min. were added 1:1 to wells containing rhPAD2 (300 ng/ml) in 100 mM Tris-HCl including various concentrations of glutathione (GSH) and 5 mM CaCl_2_. In both experiments, mAb 20B2 recognizing a citrullinated epitope on fibrinogen was used as probe. PAD activity is shown as per cent of maximal activity for each isoform, expressed as mean and range of duplicate measurements.

Using human fibrinogen coated on microtiter plates as substrate, we measured the catalytic activity of rhPAD2 or rhPAD4 in the presence of a reaction buffer containing 15 mM GSH and 5 mM calcium, previously shown to be optimal for PADs to be enzymatically active^11^. At concentrations above 40 µM, exogenously added H_2_O_2_ lowered the catalytic activity of rhPAD2 and rhPAD4 in a dose-dependent manner, which did not differ between the two PAD isoforms (Figure 1A) Half-maximal PAD activity was observed at ∼130 µM H_2_O_2_, and virtually no activity was observed at concentrations above 600 µM. At neither of concentrations used did H_2_O_2_ cause changes in fibrinogen affecting the binding of the probing antibody or potentially influential changes in pH^32^ (data not shown).

To examine if ROS are secreted by leukocytes in sufficient amounts to inhibit PAD activity, we added supernatants from PMA-stimulated or unstimulated leukocytes to rhPAD2 in reaction buffer (Figure 1(B)). More reducing agent (25 mM GSH) was required to obtain maximal rhPAD2 activity in the presence of supernatants from stimulated leukocytes than in the presence of supernatants from unstimulated leukocytes (∼15 mM GSH), suggesting that ROS secreted by activated leukocytes inhibit PAD.

### Inhibition of NOX2 enhances the activity of PAD released from activated leukocytes

**Figure 2.**
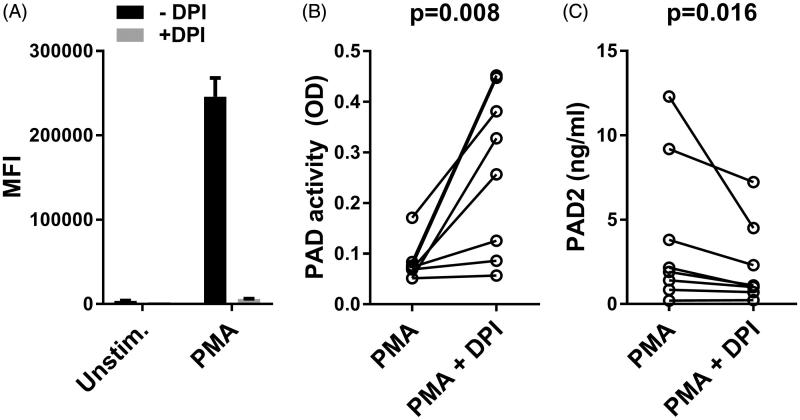
Influence of oxidation on PAD activity and amount of PAD2 released from activated leukocytes. (A) Bars show the median fluorescence intensity (MFI) of rhodamine-123 by CD15-positive, live (NIR-negative) granulocytes from two independent experiments. Leukocytes from two donors resuspended in RPMI medium containing 5% normal human serum were left unstimulated or were stimulated with PMA in the absence or presence of the NADPH oxidase inhibitor DPI. (B) Microtiter wells were coated with human fibrinogen and incubated for 3 h with PMA-stimulated leukocytes in the absence or presence of DPI, and mAb 20B2 was used as probe for citrullination. The average of duplicate measurements for each of eight donors is shown. (C) The corresponding concentration of PAD2 in the supernatants is shown.

Granulocytes are the main source of enzymatically active PAD in the vicinity of PMA-stimulated leukocytes^11^. We therefore measured H_2_O_2_ production by granulocytes using DHR-123 as probe. As expected, PMA induced extensive production of H_2_O_2_ by granulocytes, which was almost abrogated by inhibition of NOX2 (Figure 2(A) and flow cytometry histograms in Supplementary Figure S1).

The extracellular PAD activity from PMA-stimulated leukocytes was increased after DPI-mediated inhibition of the NOX2, indicating that ROS formation inactivates PAD and thereby extracellular citrullination (Figure 2(B)). The observed increase in PAD activity was not a result of increased PAD release, since a decrease was observed in the levels of extracellular PAD2 (which accounts for the major activity in the assay employed^33^) (Figure 2(C)). In agreement with previous findings^34^, the proportion of live granulocytes increased from an average of 36% to 68% after inclusion of DPI (data not shown).

### Only supraphysiological levels of ROS inhibit citrullination by PAD released from leukocytes

**Figure 3.**
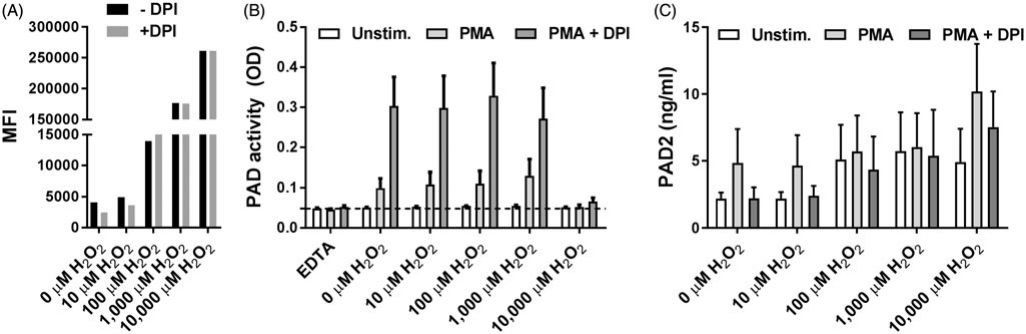
PAD activity and PAD2 release in stimulated leukocytes in the presence of H_2_O_2_. (A) Leukocytes were exposed to various concentration of H_2_O_2_ in the absence and presence of DPI. Histograms show the median fluorescence intensity (MFI) of rhodamine-123 in CD15-positive, live (NIR-negative) granulocytes. (B) Microtiter wells coated with human fibrinogen were incubated for 3 h at room temperature with unstimulated or PMA-stimulated leukocytes resuspended in RPMI medium containing 5% normal human serum with or without DPI and various concentrations of H_2_O_2_. Citrullination of fibrinogen was measured using mAb 20B2 as detecting antibody. Bars and error bars show mean and SEM of experiments involving four healthy donors. (C) PAD2 concentrations in the corresponding cell supernatants are shown.

Exogenously added H_2_O_2_ oxidized DHR-123 in a dose-dependent manner, as expected in view of its ability to diffuse over cell membranes^35^ (Figure 3(A) and flow cytometry histograms in Supplementary Figure S1). The oxidation observed in the presence of highest concentration of H_2_O_2_ tested, 10,000 µM, exceeded that observed upon stimulation with PMA (Figure 2(A)). To examine the influence of ROS on the activity of PAD released from leukocytes, we added H_2_O_2_ to unstimulated and PMA-stimulated leukocytes (Figure 3(B)). At a range of 10–1000 µM, exogenously added H_2_O_2_ had little effect on the extracellular citrullination. Endogenously produced ROS, on the other hand, mediated a much more efficient reduction in PAD activity, as demonstrated by a markedly enhanced citrullination in the presence of DPI (Figures 2(B) and 3(B)). At a concentration of 10,000 µM, however, H_2_O_2_ inhibited citrullination both in the presence and absence of DPI. This was not a result of a reduction of PAD release, since PAD2 release was slightly increased under these conditions (Figure 3(C)).

## Discussion

In view of previous findings that reduction by GSH is required for PAD activity^11^, we hypothesized that ROS would have an opposing effect. At concentrations above 40 µM, H_2_O_2_ directly inactivates the enzymatic activity of rhPAD2 and rhPAD4 reduced by GSH at a concentration of 15 mM, which can be achieved intracellularly^9^^,^^10^. Concordantly, supernatants from PMA-stimulated leukocytes inhibited rhPAD2-mediated citrullination observed at this GSH concentration, suggesting that the PMA-stimulated leukocytes have released ROS into the supernatant. Presumably neutrophils were the main contributors to the secretion of ROS^16–22^. In fact, release of H_2_O_2_ by PMA-stimulated human neutrophils has been confirmed by various methods^36–38^.

To avoid stimulation of granulocytes by handling and to mimic *in vivo* conditions, e.g. in an inflamed joint, we used unseparated leukocyte populations in this study. In keeping with the inhibitory effect of H_2_O_2_ on rhPAD, the citrullination catalysed by PAD released from activated leukocytes was inhibited by ROS produced by the PMA-stimulated leukocytes, as demonstrated by a significant increase in PAD activity after inhibition of the NOX2 with DPI. This increase occurred, even though DPI lowered PAD2 release, presumably by lowering cell death and thereby leakage of PAD2. Granulocytes are the predominant source of extracellular PAD upon stimulation with PMA^11^, and also the prime site for intracellular hypercitrullination^39^. The NOX2 generates ROS that are delivered either into the extracellular space or to intracellular compartments (reviewed in Ref. [40]), and it cannot be determined whether PAD was oxidized intracellularly, prior to release, or extracellularly in the present study. Since extracellular, matrix-bound fibrinogen was used as probe for citrullination, the increased citrullination observed following incubation of leukocytes with DPI suggests that NOX2-mediated oxidation regulates extracellular citrullination, if not also intracellular citrullination.

At a supraphysiological concentration of 10,000 µM, exogenously added H_2_O_2_ completely abrogated activity of PAD released from leukocytes. This is in line with the observation by Neeli et al. that addition of 10,000 µM H_2_O_2_ to neutrophils prevents citrullination of histone H3^24^. Concentrations of 10–1000 µM H_2_O_2_, on the other hand, promoted intracellular histone citrullination in their study^24^, but showed no effect on extracellular citrullination in the present study. Extracellular concentrations of H_2_O_2_ above 1 mM are not considered physiological achievable^41^^,^^42^, but we cannot rule out that H_2_O_2_ concentrations transiently exceed this level, which may inhibit extracellular PAD *in vivo*. It can be speculated that PAD is relatively well protected from oxidation by H_2_O_2_ within the cell, e.g. by co-localizing with GSH and catalases. In fact, intense activity of the myeloperoxidase prevents accumulation of H_2_O_2_ above 30 µM in the phagosome in spite of superoxide (O_2_^−1^) being produced at levels exceeding 1 M^43^.

Most studies find that PMA and H_2_O_2_ promote citrullination of histone H3^24^^,^^39^^,^^23^, but in some studies PMA fails to do so^44^^,^^45^. To the best of our knowledge, no other proteins than histones have been shown to be citrullinated by PAD in combination with H_2_O_2_ or other ROS. PMA-induced histone H3 citrullination can be inhibited by DPI^39^, suggesting that citrullination of histone H3 is, indeed, ROS dependent. Calcium ionophores are inducers of intracellular hypercitrullination^45^, but this citrullination can be inhibited by PMA^44^, presumably by the ROS that PMA induces. It can be speculated that the previously reported enhancing effect of ROS on histone citrullination is secondary to ROS’ facilitation of calcium transport, e.g. into the nucleus^46^^,^^47^.

Several observations indicate that RA patients have a dysregulated redox balance, e.g. increased levels of antioxidants, limited access to oxygen within the joints, and granulocytes with impaired ability to produce ROS: RA patients have increased glutathione reductase activity in synovial fluid (SF), compared to SF from reactive arthritis and osteoarthritis patients^48^, and the antioxidant thioredoxin has been reported to be increased 5-fold in SF from RA patients compared to SF from patients with osteoarthritis^49^. In accordance, SF from RA patients has higher anti-oxidative capacity than SF from osteoarthritis patients^50^, and anti-CCP positive patients’ SF has higher antioxidative capacity than that of anti-CCP negative patients^51^. As molecular oxygen (O_2_) is prerequisite for ROS production by leukocytes^52^^,^^53^, the low content of O_2_ in synovial joint fluids and tissue in RA^54^^,^^55^ presumably is reflected by sparse accumulation of H_2_O_2_. In fact, the levels of O_2_ in the joints of patients with RA are usually lower than the estimated *K*_m_ of the NOX2 at 30 µM O_2_^53^, and ROS production by phagocytes isolated from RA patients is reduced^56^^,^^57^. Thus, accumulation of H_2_O_2_ at levels capable of inhibiting PADs may not be achieved, which may cause the excessive protein citrullination in the joints of RA^27^^,^^58^.

## Conclusion

In conclusion, we demonstrate that the catalytic activity of PAD2 and PAD4 is inhibited by H_2_O_2_, and that extracellular citrullination is inhibited by ROS generated by NOX2 in activated leukocytes. This confirms our previous suggestion^11^ that the catalytic activity of PAD is enhanced in reducing conditions in vivo, and thereby is regulated by the redox balance.

## Supplementary Material

Supporting Information
