# Supplementary material for: Reactive oxygen species inhibit catalytic activity of peptidylarginine deiminase
Source: J Enzyme Inhib Med Chem. 2017 Sep 21;32(1):1203–8. doi: 10.1080/14756366.2017.1368505 (PMC6021033; doi:10.1080/14756366.2017.1368505)
Supplement: Supporting Information [file IENZ_A_1368505_SM1172.pdf]

Supporting Information

Synthesis, in vitro antitumor activity, and molecular docking study of novel 2-substituted mercapto-3-(3,4,5-trimethoxybenzyl)-4(3H)-quinazolinone analogs

Adel S. El-Azab [a,b] *, Alaa A.-M. Abdel-Aziz [a,c*], Hazem A. Ghabbour [a,c], Manal A. Al-Gendy [a]

[a]*Department of Pharmaceutical Chemistry, College of Pharmacy, King Saud University, Riyadh 11451, Saudi Arabia*

[b]*Department of Organic Chemistry, Faculty of Pharmacy, Al-Azhar University, Cairo 11884, Egypt*

[c]*Department of Medicinal Chemistry, Faculty of Pharmacy, University of Mansoura, Mansoura 35516, Egypt*

[e]*Department of Pharmaceutical Chemistry, College of Pharmacy, Sattam bin Abdulaziz University, AlKharj, Saudi Arabia*

Correspondence: College of Pharmacy, P.O. Box 2457, King Saud University, Riyadh-11451, Saudi Arabia (A. S. El-Azab, E-mail: adelazab@ksu.edu.sa, adelazaba@yahoo.com) and (A. A.-M. Abdel-Aziz, E-mail: almoenes@ksu.edu.sa, alaa_moenes@yahoo.com)

Figure Captions

Figure S1: ORTEP diagram of compound **8**. Displacement ellipsoids are plotted at the 40% probability level for non-H atoms

Figure S2: Molecular packing of titled compound viewed hydrogen bonds which are drawn as dashed lines along b axis.

Table S1: X-ray crystallographic data for compound 8.

Table S2: Geometric parameters (Å, °) of compound **8**

Table S3: Hydrogen-bond geometry (Å, °)

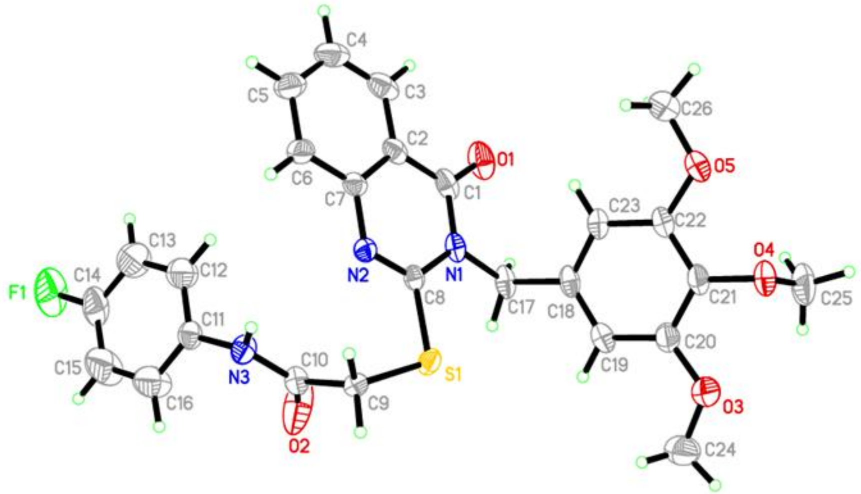

Figure S1: ORTEP diagram of compound **8**. Displacement ellipsoids are plotted at the 40% probability level for non-H atoms.

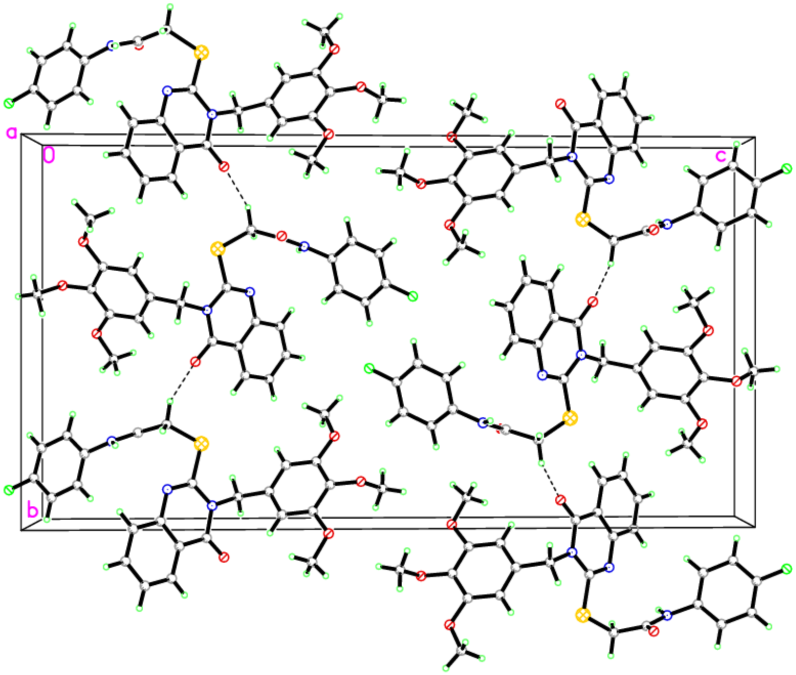

Figure S2: Molecular packing of titled compound viewed hydrogen bonds which are drawn as dashed lines along *b* axis.

**Crystal data**	
Chemical formula	$C_{26}H_{24}FN_3O_5S$
Mr	509.54
Crystal system, space group	Monoclinic, $P2_1/n$
Temperature (K)	293
a, *b*, *c* (Å)	4.6461 (2), 16.7264 (6), 31.2525 (10)
β (°)	92.301 (1)
V (Å^3)	2426.75 (16)
Z	4
Radiation type	Mo *Kα*
μ (mm^{-1})	0.18
Crystal size (mm)	0.42 × 0.17 × 0.05
**Data collection**	
Diffractometer	Bruker APEX-II D8 venture diffractometer
Absorption correction	Multi-scan<br>SADABS Bruker 2014
Tmin, Tmax	0.927, 0.991
No. of measured, independent and observed [I > 2σ(I)] reflections	60929, 9710, 5125
R_{int}	0.058
**Refinement**	
$R[F^2 > 2\sigma(F^2)]$, $wR(F^2)$, S	0.074, 0.221, 1.02
No. of reflections	9710
No. of parameters	332
No. of restraints	0
H-atom treatment	H atoms treated by a mixture of independent and constrained refinement
$\Delta\rho_{max}$, $\Delta\rho_{min}$ (e Å^{-3})	0.58, −0.36

Table S1: X-ray crystallographic data for compound **8**.

Table S2 Geometric parameters (Å, °)

S1—C8	1.768 (2)	C9—H9B	0.9900
S1—C9	1.800 (2)	C11—C16	1.348 (4)
F1—C14	1.363 (3)	C11—C12	1.361 (4)
O1—C1	1.225 (3)	C12—C13	1.397 (4)
O2—C10	1.195 (3)	C12—H12A	0.9500
O3—C20	1.363 (3)	C13—C14	1.337 (5)
O3—C24	1.401 (4)	C13—H13A	0.9500
O4—C21	1.376 (3)	C14—C15	1.334 (5)
O4—C25	1.423 (5)	C15—C16	1.385 (4)
O5—C22	1.365 (3)	C15—H15A	0.9500
O5—C26	1.416 (4)	C16—H16A	0.9500
N1—C8	1.386 (2)	C17—C18	1.514 (3)
N1—C1	1.401 (3)	C17—H17A	0.9900
N1—C17	1.476 (3)	C17—H17B	0.9900
N2—C8	1.286 (2)	C18—C19	1.379 (3)
N2—C7	1.385 (3)	C18—C23	1.383 (3)
N3—C10	1.315 (3)	C19—C20	1.397 (3)
N3—C11	1.428 (3)	C19—H19A	0.9500
N3—H1N3	0.79 (3)	C20—C21	1.380 (3)
C1—C2	1.449 (4)	C21—C22	1.385 (4)
C2—C7	1.397 (3)	C22—C23	1.396 (3)
C2—C3	1.407 (3)	C23—H23A	0.9500

C3—C4	1.368 (4)	C24—H24A	0.9800
C3—H3A	0.9500	C24—H24B	0.9800
C4—C5	1.381 (4)	C24—H24C	0.9800
C4—H4A	0.9500	C25—H25A	0.9800
C5—C6	1.378 (3)	C25—H25B	0.9800
C5—H5A	0.9500	C25—H25C	0.9800
C6—C7	1.403 (3)	C26—H26A	0.9800
C6—H6A	0.9500	C26—H26B	0.9800
C9—C10	1.515 (3)	C26—H26C	0.9800
C9—H9A	0.9900		
C8—S1—C9	99.70 (9)	C12—C13—H13A	120.9
C20—O3—C24	119.3 (2)	C15—C14—C13	122.3 (3)
C21—O4—C25	113.2 (3)	C15—C14—F1	119.8 (3)
C22—O5—C26	118.1 (2)	C13—C14—F1	117.7 (3)
C8—N1—C1	120.50 (18)	C14—C15—C16	119.1 (3)
C8—N1—C17	121.65 (19)	C14—C15—H15A	120.5
C1—N1—C17	117.73 (18)	C16—C15—H15A	120.5
C8—N2—C7	118.18 (17)	C11—C16—C15	120.6 (3)
C10—N3—C11	123.51 (19)	C11—C16—H16A	119.7
C10—N3—H1N3	111 (2)	C15—C16—H16A	119.7
C11—N3—H1N3	122 (2)	N1—C17—C18	114.64 (17)
O1—C1—N1	120.2 (2)	N1—C17—H17A	108.6
O1—C1—C2	124.3 (2)	C18—C17—H17A	108.6
N1—C1—C2	115.48 (18)	N1—C17—H17B	108.6
C7—C2—C3	119.8 (2)	C18—C17—H17B	108.6
C7—C2—C1	119.1 (2)	H17A—C17—H17B	107.6
C3—C2—C1	121.1 (2)	C19—C18—C23	120.20 (19)
C4—C3—C2	119.7 (3)	C19—C18—C17	119.6 (2)
C4—C3—H3A	120.2	C23—C18—C17	120.0 (2)
C2—C3—H3A	120.2	C18—C19—C20	119.9 (2)
C3—C4—C5	120.6 (2)	C18—C19—H19A	120.1
C3—C4—H4A	119.7	C20—C19—H19A	120.1
C5—C4—H4A	119.7	O3—C20—C21	114.8 (2)
C6—C5—C4	121.2 (3)	O3—C20—C19	124.9 (2)
C6—C5—H5A	119.4	C21—C20—C19	120.3 (2)
C4—C5—H5A	119.4	O4—C21—C20	120.3 (2)
C5—C6—C7	119.2 (2)	O4—C21—C22	120.0 (2)
C5—C6—H6A	120.4	C20—C21—C22	119.7 (2)
C7—C6—H6A	120.4	O5—C22—C21	115.33 (19)
N2—C7—C2	121.9 (2)	O5—C22—C23	124.4 (2)
N2—C7—C6	118.49 (19)	C21—C22—C23	120.2 (2)
C2—C7—C6	119.6 (2)	C18—C23—C22	119.7 (2)
N2—C8—N1	124.81 (19)	C18—C23—H23A	120.1
N2—C8—S1	118.92 (14)	C22—C23—H23A	120.1
N1—C8—S1	116.27 (14)	O3—C24—H24A	109.5
C10—C9—S1	111.56 (13)	O3—C24—H24B	109.5
C10—C9—H9A	109.3	H24A—C24—H24B	109.5
S1—C9—H9A	109.3	O3—C24—H24C	109.5
C10—C9—H9B	109.3	H24A—C24—H24C	109.5

S1—C9—H9B	109.3	H24B—C24—H24C	109.5
H9A—C9—H9B	108.0	O4—C25—H25A	109.5
O2—C10—N3	121.9 (2)	O4—C25—H25B	109.5
O2—C10—C9	121.2 (2)	H25A—C25—H25B	109.5
N3—C10—C9	116.88 (17)	O4—C25—H25C	109.5
C16—C11—C12	118.9 (2)	H25A—C25—H25C	109.5
C16—C11—N3	121.9 (2)	H25B—C25—H25C	109.5
C12—C11—N3	119.0 (2)	O5—C26—H26A	109.5
C11—C12—C13	120.6 (3)	O5—C26—H26B	109.5
C11—C12—H12A	119.7	H26A—C26—H26B	109.5
C13—C12—H12A	119.7	O5—C26—H26C	109.5
C14—C13—C12	118.1 (3)	H26A—C26—H26C	109.5
C14—C13—H13A	120.9	H26B—C26—H26C	109.5
C8—N1—C1—O1	−178.69 (19)	C16—C11—C12—C13	3.4 (6)
C17—N1—C1—O1	−2.6 (3)	N3—C11—C12—C13	178.4 (3)
C8—N1—C1—C2	2.5 (3)	C11—C12—C13—C14	1.9 (7)
C17—N1—C1—C2	178.61 (16)	C12—C13—C14—C15	−6.5 (7)
O1—C1—C2—C7	179.4 (2)	C12—C13—C14—F1	178.9 (4)
N1—C1—C2—C7	−1.8 (3)	C13—C14—C15—C16	5.5 (7)
O1—C1—C2—C3	−1.0 (3)	F1—C14—C15—C16	−180.0 (4)
N1—C1—C2—C3	177.80 (19)	C12—C11—C16—C15	−4.5 (6)
C7—C2—C3—C4	−0.6 (3)	N3—C11—C16—C15	−179.3 (4)
C1—C2—C3—C4	179.8 (2)	C14—C15—C16—C11	0.2 (8)
C2—C3—C4—C5	0.1 (4)	C8—N1—C17—C18	−92.6 (2)
C3—C4—C5—C6	0.7 (4)	C1—N1—C17—C18	91.3 (2)
C4—C5—C6—C7	−1.0 (4)	N1—C17—C18—C19	131.2 (2)
C8—N2—C7—C2	1.0 (3)	N1—C17—C18—C23	−54.3 (3)
C8—N2—C7—C6	−178.83 (17)	C23—C18—C19—C20	−1.6 (3)
C3—C2—C7—N2	−179.52 (19)	C17—C18—C19—C20	172.9 (2)
C1—C2—C7—N2	0.1 (3)	C24—O3—C20—C21	−170.5 (3)
C3—C2—C7—C6	0.3 (3)	C24—O3—C20—C19	10.6 (4)
C1—C2—C7—C6	179.95 (18)	C18—C19—C20—O3	178.2 (2)
C5—C6—C7—N2	−179.7 (2)	C18—C19—C20—C21	−0.7 (4)
C5—C6—C7—C2	0.4 (3)	C25—O4—C21—C20	78.8 (3)
C7—N2—C8—N1	−0.3 (3)	C25—O4—C21—C22	−101.8 (3)
C7—N2—C8—S1	179.13 (13)	O3—C20—C21—O4	3.0 (4)
C1—N1—C8—N2	−1.5 (3)	C19—C20—C21—O4	−178.0 (2)
C17—N1—C8—N2	−177.49 (17)	O3—C20—C21—C22	−176.3 (2)
C1—N1—C8—S1	179.00 (14)	C19—C20—C21—C22	2.7 (4)
C17—N1—C8—S1	3.0 (2)	C26—O5—C22—C21	179.9 (2)
C9—S1—C8—N2	8.14 (17)	C26—O5—C22—C23	1.7 (4)
C9—S1—C8—N1	−172.35 (14)	O4—C21—C22—O5	0.0 (4)
C8—S1—C9—C10	69.34 (15)	C20—C21—C22—O5	179.3 (2)
C11—N3—C10—O2	−10.0 (4)	O4—C21—C22—C23	178.2 (2)
C11—N3—C10—C9	171.7 (2)	C20—C21—C22—C23	−2.4 (4)
S1—C9—C10—O2	50.1 (3)	C19—C18—C23—C22	1.8 (3)
S1—C9—C10—N3	−131.64 (18)	C17—C18—C23—C22	−172.64 (19)
C10—N3—C11—C16	67.7 (4)	O5—C22—C23—C18	178.3 (2)
C10—N3—C11—C12	−107.2 (3)	C21—C22—C23—C18	0.2 (3)

Table S3: Hydrogen-bond geometry (Å, °)

**D—H···A**	**D—H**	**H···A**	**D···A**	**D—H···A**
N3—H1N3···O2^{i}	0.79 (3)	2.07 (3)	2.818 (3)	158 (3)
C9—H9A···O1^{ii}	0.9900	2.3500	3.196 (3)	143.00
C9—H9B···O2^{i}	0.9900	2.3100	3.171 (3)	144.00
C23—H23A···O1	0.9500	2.4600	3.178 (3)	132.00
Symmetry codes: (i) x+1, y, z; (ii) −x+1/2, y+1/2, −z+3/2.				
